# Early land plants: Plentiful but neglected nutritional resources for herbivores?

**DOI:** 10.1002/ece3.9617

**Published:** 2022-12-12

**Authors:** Audrey Duhin, Ricardo A. R. Machado, Ted C. J. Turlings, Gregory Röder

**Affiliations:** ^1^ Laboratory of Fundamental and Applied Research in Chemical Ecology, Institute of Biology University of Neuchâtel Neuchâtel Switzerland; ^2^ Experimental Biology Research Group, Institute of Biology University of Neuchâtel Neuchâtel Switzerland

**Keywords:** early land plants, ferns, generalist herbivores, insect, mosses, nutritive content, snail

## Abstract

Plants and herbivores have been engaged in a co‐evolutionary arms race for millions of years, during which plants evolved various defenses and other traits to cope with herbivores, whereas herbivores evolved traits to overcome the plants' resistance strategies. Herbivores may also avoid certain plants merely because these lack suitable nutrients for their development. Interestingly, the number of herbivores that attack individual early land plants like mosses and ferns is quite low. Among others, poor nutrient quality has been hypothesized to explain the apparent low herbivory pressure on such plants but still waits for scientific evidences. Here, the nutritive suitability of representative mosses and liverworts (bryophytes) and ferns (pteridophytes) for herbivores was investigated using feeding assays combined with quantifications of nutrients (proteins, amino acids, and sugars). Growth and survival of two polyphagous herbivores, a caterpillar and a snail, were monitored when fed on 15 species of bryophytes and pteridophytes, as well as on maize (*Zea mays*, angiosperm) used as an external indicative nutritional resource. Overall, our results show that the poor performance of the herbivores on the studied early land plants is not correlated with nutritional quality. The growth and performance of snails and caterpillars fed with these plants were highly variable and independent of nutrient content. These findings arguably dismiss the poor nutrient quality hypothesis as the cause of herbivory deficit in bryophytes and pteridophytes. They suggest the possible presence of early resistance traits that have persisted all through the long evolutionary history of plant–herbivore interactions.

## Graphical abstract

In this paper, we explore the unknown reason(s) why herbivores rarely seem to attack early land plants like mosses and ferns. Our study, in which we looked at the performance of an arthropod and a gastropod on 15 species of such plants, yields surprising results that disqualify some of the common notions about early land plants.

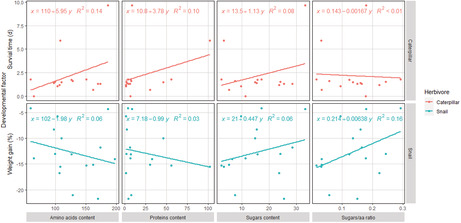

## 1 INTRODUCTION

Evolutionary studies have shown that bryophytes are the earliest plants that moved from their aquatic habitats to colonize terrestrial ecosystems during Ordovician (ca. 449 million years ago (Ma); Clarke et al., 2011). The first land plants had to face and adapt to many new stress factors such as ultraviolet radiation (UV) from direct sunlight, risk of desiccation, and gravity (He et al., 2013; Horn, 1971; Rozema et al., 2002). Bryophytes are divided into three groups: Hepaticophyta (liverworts), Bryophyta (mosses), and Anthocerotophyta (hornworts; Shaw & Renzaglia, 2004). Later, the first vascular plants evolved in the paraphyletic pteridophytes group (sensu Pryer et al. (2004)) with the clubmosses (Lycophyta), and both the horsetails and the ferns (Monilophyta). Nowadays, seed plants (Spermatophyta) gather all the gymnosperms and angiosperms (Nickrent et al., 2000; Qiu et al., 2006; Ruhfel et al., 2014).

Herbivory is a ubiquitous phenomenon that affects most seed plants (Marquis, 1992). By contrast, bryophytes and pteridophytes are generally considered to be largely spared from such antagonistic interactions (Cooper‐Driver, 1978; Gerson, 1969; Glime, 2006; Hendrix, 1977; Soo Hoo & Fraenkel, 1964). Although they lack tough tissues like wood or cork, these apparently fragile and readily accessible plants are mostly avoided by modern herbivores. This may explain why few studies have been carried out on the consumption of mosses and ferns by herbivores (Haines & Renwick, 2009). For decades, it has been hypothesized that early land plants are unsuitable hosts for herbivorous insects, lacking crucial nutrients (namely the poor nutrient quality hypothesis, Ehrlich & Raven, 1964; Hernick et al., 2008; Prins, 1982). Deterring secondary metabolites and digestive chemical or physical inhibitors would be the alternative hypotheses explaining this apparent dislike (Haines & Renwick, 2009). In 1964, Ehrlich and Raven stated that among herbivorous lepidopteran larvae, none is known to feed on bryophytes, nor on ferns, before mentioning the fern eaters *Papaipema* moths (Noctuidae; see page 598 in Ehrlich & Raven, 1964). Years later, studies on early land plants reported that various herbivores, including mammals, birds, gastropods, and arthropods can actually feed on bryophytes (Davidson et al., 1990; Fang & Zhu, 2013; Glime, 2017; Maciel‐Silva & Santos, 2011; Singer & Mallet, 1986) and pteridophytes (Hamm & Fordyce, 2016; Schneider, 2016). Some insects have even been shown to specialize on mosses, like weevils from the tribe Ectemnorhinini (Coleoptera: Curculionidae; Chown, 1990), larvae of some snipe flies (Diptera: Rhagionidae; Imada & Kato, 2016), or crane flies species (Diptera: Tipulidae; Freeman, 1967). In this context, Smith et al. (2001) evaluated the development of crane flies on five different moss species, finding that all larvae gained weight and seemed to choose which plants were of the best quality. Yet they concluded that herbivores probably select bryophytes as shelter rather than as food, because crane fly larvae still preferred angiosperm leaves, if available. Noticeable exceptions to fill the gap in our knowledge on herbivory in early plants were works of Markham et al. (2006), which assessed protein‐based defenses against phytophagous insects in several ferns and mosses, of Haines and Renwick (2009), which suggested that some preingestive mechanisms might be crucial in deterring herbivory on mosses rather than nutrient poverty, and both of Hendrix and Marquis (1983) and Patra and Bera (2007) showing that damages caused by herbivory in three, and 11, respectively, tropical fern species can be compared to that found for angiosperms. In cold and heathland environments, only generalist herbivores have been observed to feed on mosses (Butet, 1990; Crafford & Chown, 1991).

It is relevant to point out that mosses lack hard supporting sclerenchyma and resulting stiff vascular tissues (i.e., phloem and xylem). Despite some fossil‐based evidence for arthropod herbivory on liverworts from the Middle Devonian (Labandeira et al., 2014), it has been suggested that these soft tissues are less prompt to fossilize, compared to that of vascular plants (Kenrick et al., 2012), making difficult to properly document the 400 Ma old interactions between mosses and herbivores. Records of leaf damage on fern fossils seem to be more common. For clubmosses and ferns, Paleozoic indications of herbivory can be seen from the Late Silurian to Early Devonian (417 to 403 Ma ago; Labandeira, 2007) or from Triassic (252 to 201 Ma ago; Imada et al., 2022) and the Late Miocene (10.29 to 5.27 Ma ago), which has firmly been attributed to insects (Robledo et al., 2015). Nowadays, few insects are specialized in ferns, but some sawfly species (Hymenoptera: Tenthredinidae) are known to lay their eggs in new fern fronds, where their larvae can feed on fresh tissues right after hatching (Schreiner et al., 1984). Mostly, ferns, like mosses, are considered to be unsuitable host plants, although few studies only really focused on their nutritional value (Buckingham et al., 1978).

Regarding the chemical defenses in early land plants, liverworts have been shown to contain defense compounds like oxylipins (Croisier et al., 2010; Ponce de Leon et al., 2015; Rempt & Pohnert, 2010), terpenes, aromatics (He et al., 2013), and sesquiterpene lactones (Asakawa & Takemoto, 1979; Knoche et al., 1969). In mosses, phenols have been found to accumulate in gametophytic cell walls (Davidson et al., 1989, 1990) and show repellent effects on gastropods and isopods (Glime, 2006). The aquatic moss *Fontinalis novae‐angliae* contains fatty acids that repel insects (Parker et al., 2007). In pteridophytes, some clubmosses rely on alkaloids for chemical defense (Aver & Trifonov, 1994; Kitajima & Takayama, 2011). Ferns also contain phenols (Bohm, 1968; Bohm & Tryon, 1967), as well as flavonoids (Star & Mabry, 1971). The bracken fern *Pteridium aquilinum* contains cyanogenic glucosides with proven negative effects on insect development (Schreiner et al., 1984). All these defenses suggest that pteridophytes might be chemically protected; however, the exact influence on herbivores was rarely studied.

If present, trichomes seem to be the main physical defensive structures in bryophytes and pteridophytes, but their exact functions against herbivores still need to be studied. As well, some fern species are known to use extrafloral nectaries recruiting ants for protection against herbivores (Heads & Lawton, 1984; Koptur et al., 1998).

Surprisingly, the longstanding cohabitation of herbivores with early plants does not seem to have allowed a frequent specialization on this plentiful resource. The intriguing relationships between these plants and herbivores remain poorly understood. Further insight into these relationships could contribute to a better understanding of the early evolution of plant defenses and how they may have been maintained and adapted to changing biotic and abiotic environments (Markham et al., 2006).

Combining feeding experiments and nutritional quality assessments, our study aims to test the hypothesis that bryophytes and pteridophytes are of poor nutritional quality, which could explain, for a part, the possible low herbivory pressure on these plants. Both the survival and developmental performance of two polyphagous herbivores were monitored in feeding experiments using representative species of mosses, liverworts, and ferns, as well as one angiosperm species (maize, *Zea mays*), used as an external indicative nutritional resource. The prime objective of assessing the nutritive suitability of a representative panel of early land plants for generalist herbivores excludes an exhaustive comparison of the nutritional values shown by all the major plant taxonomic groups. In this context, sugar, protein, and amino acid contents of leaves were quantified with chemical analyses. By linking herbivore performance and plant nutritional quality, this study offers a general overview of the early land plants' suitability for the two herbivores. Our results bring to the fore that the observed herbivores' performances are not driven by nutritional quality, predicting a more important role for chemical or physical defensive mechanisms than commonly assumed in both bryophytes and pteridophytes.

## 2 MATERIAL AND METHODS

### 2.1 Plants

Wild plants were collected in and near the canton of Neuchâtel (Switzerland). If not available in their natural habitat, they were grown in the botanical garden (Jardin Botanique, Neuchâtel) or purchased in a specialized garden center (Flower Market Dietrich, GmbH). Two weeks before each experiment, all plants were acclimatized at the University of Neuchâtel in a plant growth chamber (CLF Plant Climatics) with the following conditions: 15°C; 70% RH; day/night photoperiod of 12:12 h (light:dark). All plant species used for feeding experiments and chemical analyses belong to the following three groups: (1) bryophytes with two liverworts species (*Marchantia polymorpha* and *Riccardia chamedryfolia*) and five mosses species (*Fontinalis antipyretica, Rhytidiadelphus triquetrus, Pseudocleropodium purum, Ctenidium molluscum*, and *Anomodon viticulosus*); (2) pteridophytes including one clubmoss species (*Lycopodium annotinum*), seven monilophytes (one horsetail: *Equisetum scirpoides*; and 6 ferns: *Salvinia natans, Adiantum venustum, Asplenium trichomanes, Polypodium vulgare, Polystichum aculeatum*, and *Dryopteris filix‐mas*); and (3) one external indicative angiosperm species with maize (*Zea mays* var. Delprim, 3 weeks old after germination, 3 leaves stage, grown in the plant growth chamber under the same conditions as other plants).

### 2.2 Herbivores

The two polyphagous herbivores used were second instar caterpillars of the beet armyworm *Spodoptera exigua* (Insecta, Lepidoptera: Noctuidae) reared at the University of Neuchâtel, and wild adults of *Cochlicella barbara* snail (Gastropoda, Pulmonata: Helicidae) collected near Montpellier (France). They all were kept under controlled conditions (23 ± 1°C; day/night photoperiod of 12:12 h) in growth chambers and were fed either with a lepidopteran artificial diet for caterpillars or with fresh lettuce for snails aiming to avoid any habituation for the plants tested.

### 2.3 Feeding and performance bioassays

During each trial, one individual caterpillar or snail was placed in a plastic box (5 cm diameter; 2 cm height) and provided with one of the tested fresh food ad libitum (no‐choice food test). For each plant species, 4 distinct colonies (mosses) or individual shoots (ferns) were used as food providers. A total of 40 herbivores (20 caterpillars and 20 snails) were allowed to feed on one of the 16 diet types during the bioassays. Each herbivore was weighed every 3 days and the developmental stage of the *S. exigua* caterpillars was recorded (i.e., larva, pupa, or imago). The experiment was carried out until caterpillars died or when adults emerged. The *C. barbara* snails were allowed to feed for 33 consecutive days before ending the assay. To maintain proper humidity levels a moistened piece of filter paper, for the caterpillars, or a piece of wet plastic sponge, for the snails, was placed in the plastic boxes. The bioassays were conducted under controlled laboratory conditions (artificial light with a 12:12 h (light:dark) cycle, constant 23 ± 1°C temperature). Aiming to verify that the experimental setup cannot by itself negatively influence the performances of herbivores, a commercial artificial diet designed for caterpillar rearing was added as a control, leading to a maximum of 17 different diets during the feeding and performance bioassays. The nutritive content of this artificial diet was not assessed for further comparison.

### 2.4 Nutrients quantification

Each class of nutrients (i.e., proteins, amino acids, and sugars) was measured on four different individual colonies (mosses) or shoots (ferns) per plant species (*n* = 4). For each entity, the average values from three technical replicates were combined as one biological replicate, which was used in all further statistics.

### 2.5 Proteins

Soluble proteins were extracted from 20 mg of fresh leaves using 400 μl of an SDS buffer (sodium dodecyl sulfate 2%), Tris HCl (hydrochloride) (pH 8) 100 mM, NaF (sodium fluoride) 10 mM and PIC reagents (Paired‐Ion Chromatography reagents 2.5 mM diluted in water). Samples were incubated for 30 min at 37°C in a dry bath. Then, extracts were centrifuged for 15 min at maximum speed at room temperature (22°C). To quantify proteins, 1 to 6 μl of the resulting supernatants (depending on the concentration) were added to 1 ml of the reactant mixture from the BCA (bicinchoninic acid) Protein Assay Kit (Pierce, Thermo Scientific) and incubated again for 30 min at 37°C. For quantification, the absorbance of the solution was measured at 562 nm, then compared with a calibration curve prepared with pure BSA (bovine serum albumin). Minor adjustments in terms of extract quantities were made in order to use the classic protocol developed for angiosperms by Stout and Al‐Niemi (2002). The reproducibility of the method was validated by tri‐replicates on the same sample with a coefficient of variation (or relative standard deviation) kept under 15%. As described above, three technical replicates per sample were analyzed.

### 2.6 Total amino acids

Chemical analyses of total amino acids (i.e., free and those fixed in proteins) were carried out at the Service “Biomass and Green Technologies” (University of Liège—Gembloux Agro‐Bio Tech), following the method described in Vanderplanck et al. (2013). One milliliter of hydrolysis solution (6 N HCl, 0.1% phenol, and 500 μM norleucine) was added to 3–5 mg (dry weight) of plant material. The tube was placed under nitrogen for 1 min to avoid methionine degradation and then incubated for 24 h at 110°C. The hydrolysate was evaporated until dryness under vacuum in a boiling bath at 100°C. Afterward, 1 ml of sodium citrate buffer at pH 2.2 was added. The sample solution was mixed and poured into an HPLC vial after filtration (0.2 μm). Each amino acid was measured separately with an ion exchange chromatography (Biochrom 20 Plus Amino Acid analyzer). A postcolumn ninhydrin reaction produced colored derivatives, which were monitored via a UV detector, with norleucine used as the internal standard. Using this method, 16 amino acids were quantified: Asp, Thr, Ser, Glu, Pro, Gly, Ala, Cys, Val, Ile, Leu, Tyr, Phe, His, Lys, and Arg. As for the proteins, three technical replicates per sample were analyzed.

### 2.7 Free and stored sugars

Extraction and quantification of both free (glucose, fructose, sucrose) and stored sugars (starch) were carried out following the method of Machado et al. (2013), with minor adaptations in the incubation steps. Soluble sugars were extracted from plant tissues using 80% (v/v) ethanol, followed by an incubation step (15 min at 80°C), with regular shaking. Pellets were re‐extracted twice with 50% (v/v) ethanol (15 min at 80°C). Supernatants from all extraction steps were pooled together. Afterward, sucrose, glucose, and fructose were quantified enzymatically as described by Velterop and Vos (2001). The remaining pellets were used for an enzymatic determination of starch (Smith & Zeeman, 2006). As for the other studied nutrients, three technical replicates per sample were analyzed.

### 2.8 Statistical analysis

Statistical analyses were carried out in R studio (R version 3.4.3). For leaves protein and total amino acid contents, one‐way ANOVAs were performed after log(x ± α) transformation of the raw data. The logtrans function (package MASS) was used to determine optimized (α) for each variable. Tukey post hoc tests (honestly significant difference) were used hereafter to know which pairs were significantly different, and lettering was added to statistically sort the plant species onto the figures. When ANOVA assumptions failed, like for the relative amounts of amino acid and total sugar contents, nonparametric Kruskal–Wallis tests were carried out. Both perMANOVA (permutational multiple analysis of variance) and pairwise analyses were used for detailed comparisons between amino acid contents in the plant species (Anderson, 2001). Aiming to highlight likely differences between all the studied plants, principal component analyses (PCA) were carried out on amino acids and sugars. Finally, linear regressions were realized between every nutrient parameter and the developmental performances of the two generalist herbivores, with coefficients of determination (*R* ^2^) calculated with the least square method.

## 3 RESULTS

### 3.1 Feeding and performance bioassays

**FIGURE 1:**
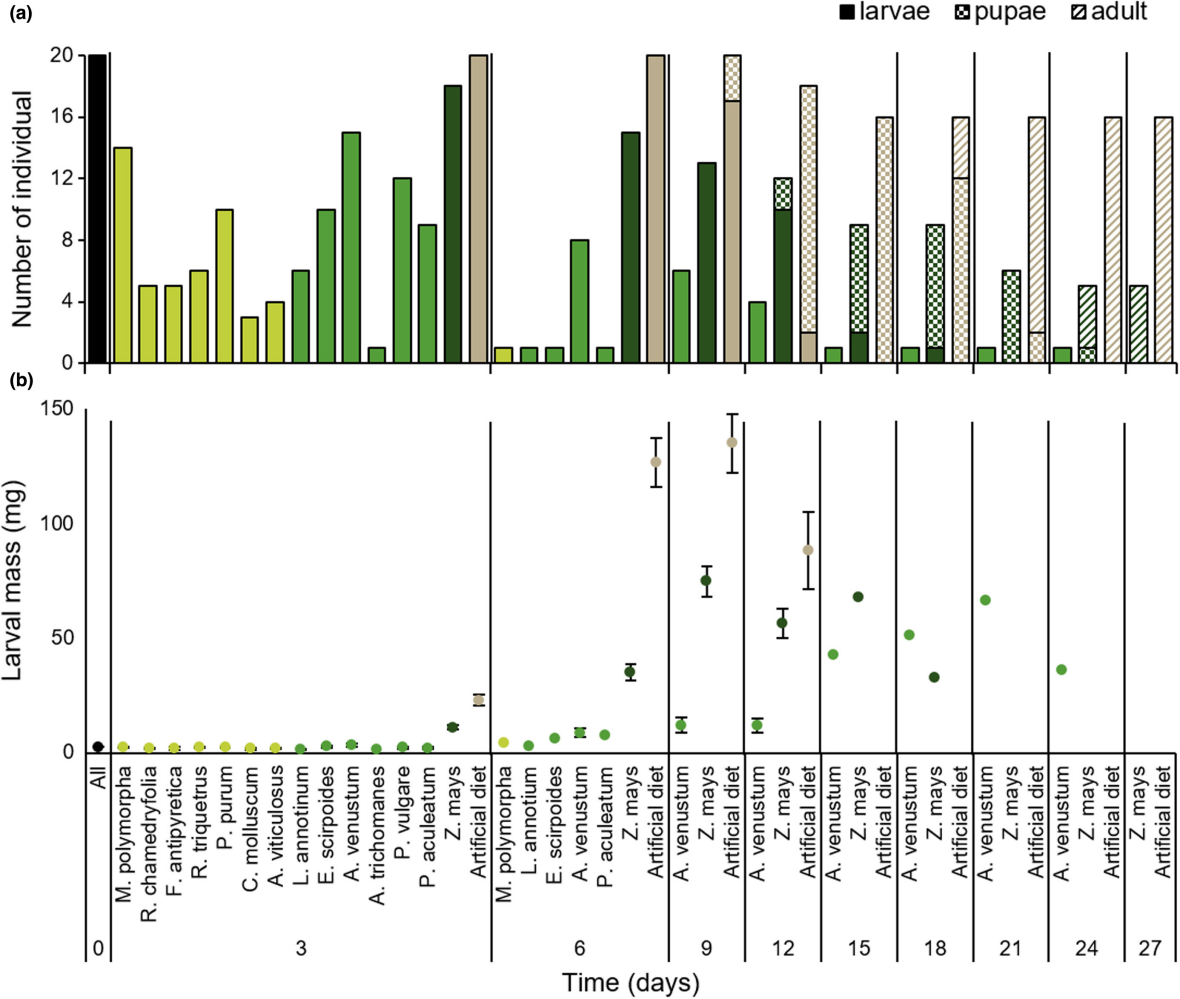
(a) Survival and developmental stage reached, and (b) larval mass monitoring of *Spodoptera exigua* caterpillars fed on primitive plants, maize, or artificial diet. Tissue of one of the 7 bryophytic (light green), 8 pteridophytic (middle green), maize (*Zea mays*, dark green) plant, or artificial diet (brown) was used to feed 20 caterpillars during the entire assays. Monitoring of individual larval mass and developmental stage was carried out every 3 days. The last adult moths had emerged from pupae on day 27, then the assays were ended. When all caterpillars assigned to one treatment died, the corresponding plant species in the figure is not shown at the next time point anymore.

Compared with *S. exigua* caterpillars fed on both artificial diet and maize plant, caterpillars fed on early land plants did not survive or develop properly (Figure 1a,b). This was highly consistent, with only one species of fern (*A. venustum*) still hosting living caterpillars after 9 days, whereas none survived beyond day 24 (Figure 1a). All caterpillars fed on primitive plants died before pupating, while 25% fed on maize (*Z. mays*) and 80% on artificial diet achieved metamorphosis into moths. Except for the only individual able to survive on *A. venustum*, all the caterpillars that gained weight were fed on maize or artificial diet, with a typical weight loss right before pupation (Figure 1b).

**FIGURE 2:**
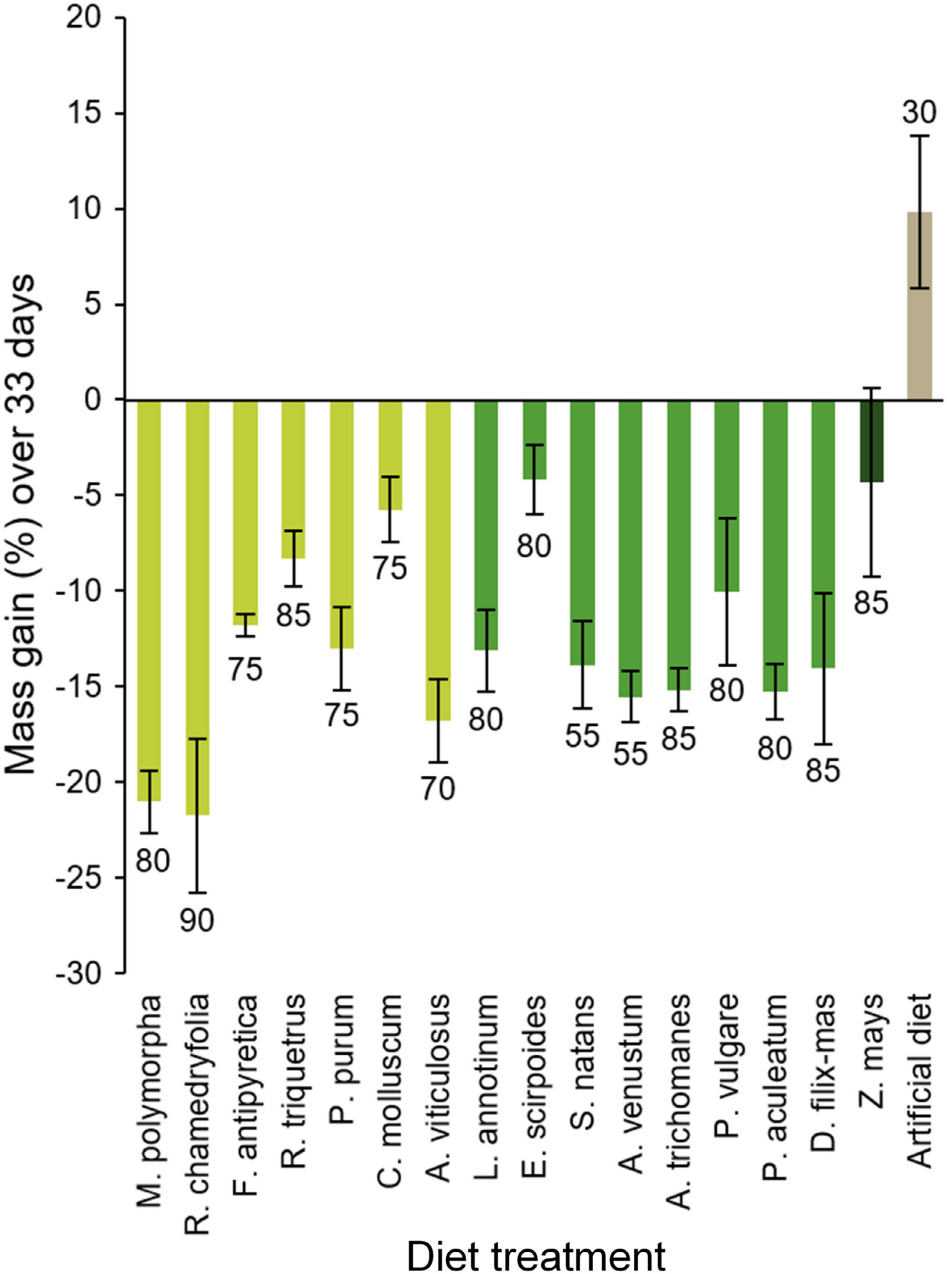
Mass gain/loss (in %) for *Cochlicella barbara* snails, fed on primitive plants, maize, or artificial diet. Tissue of one of the 7 bryophytic (light green), 8 pteridophytic (middle green), maize (*Zea mays*, dark green) plant, or artificial diet (brown) were provided during 33 consecutive days. The number next to each bar is the percent of survival after 33 days.

The polyphagous *C. barbara* snails lost weight and suffered some mortality when fed on the different plants. No exact food intake was assessed, while snails visually fed scarcely on the plants. The mass lost across time was the lowest for snails fed with horsetail (*E. scirpoides*) and maize leaves (Figure 2), whereas the most severe mass decreases were observed on liverworts species (Figure 2, *M. polymorpha* and *R. chamedryfolia*). Overall, we observed high variability in snail performances within and between the different taxonomical plant groups used as diet. While snails were able to gain mass only when fed on artificial diet, this resource, however, triggers the worst survival rate (30%), compared with all the other plant diets (survival rate from 55% to 90%).

Taken together, the feeding assays confirmed that generalist caterpillars and snails perform poorly when fed on the 15 bryophytes or pteridophytes included in the study.

### 3.2 Nutrients quantification

#### 3.2.1 Proteins

**FIGURE 3:**
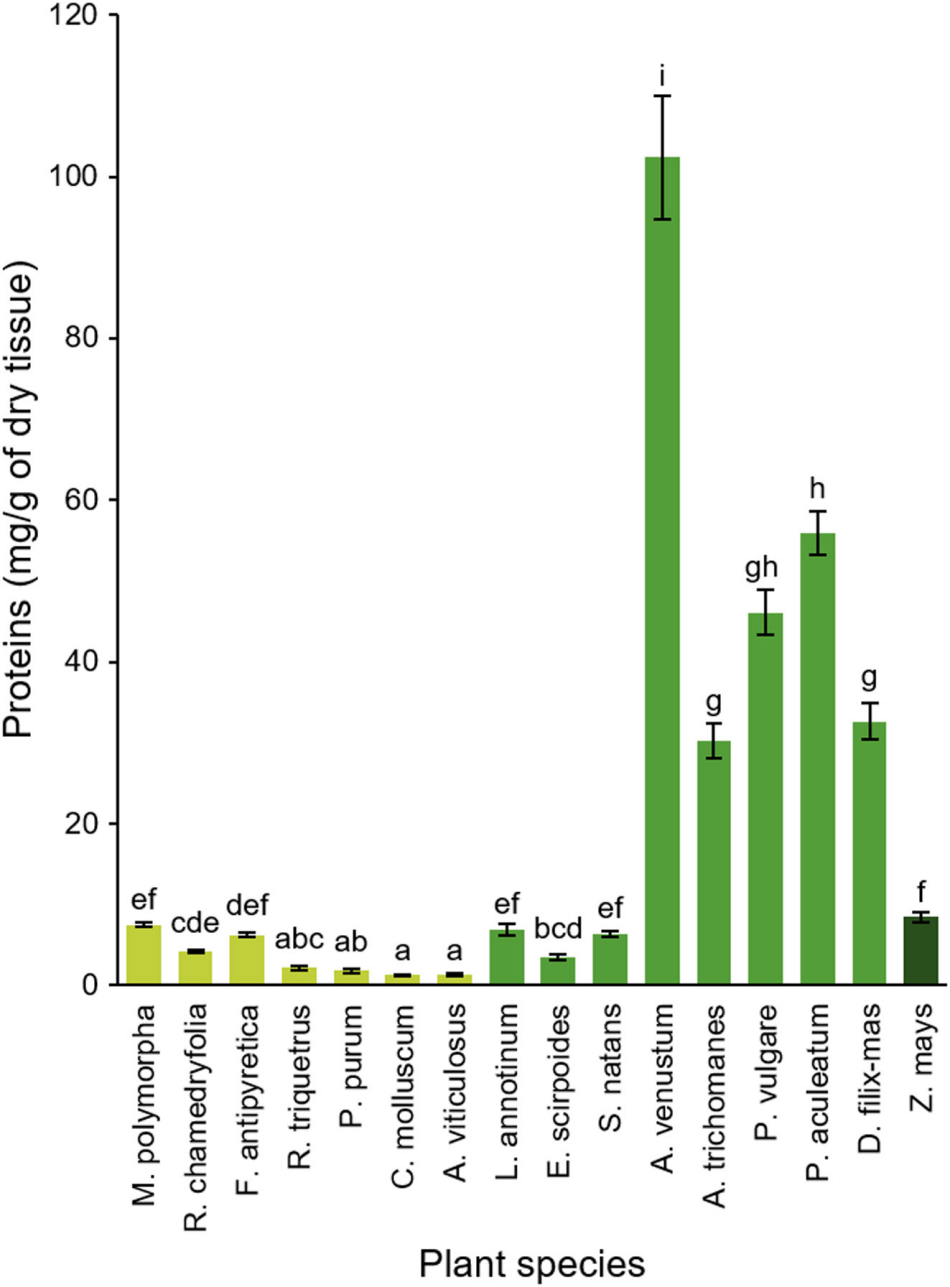
Leaf protein contents (mean ± SE, *n* = 4 for each plant species) quantified in primitive plants and maize. Tissue of one of the 7 bryophytic (light green), 8 pteridophytic (middle green), and maize (*Zea mays*, dark green) plants were analyzed. Letters above bars indicate statistical differences according to post‐ANOVA Tukey test results performed with log‐transformed data (ANOVA results: *F*_(15;48)_ = 170.2, *p*‐value < .001).

Plants showed significant differences in protein contents (*F*_(15;48)_ = 170.2, *p*‐value < .001; Figure 3). In the liverwort, *M. polymorpha* proteins showed similar quantities as those found in maize plants, whereas in *R. chamedryfolia* they were lower. Moss species contain lower amounts of proteins compared with maize, except for the aquatic *F. antipyretica*. In pteridophytes, the horsetail *E. scirpoides* had the lowest protein level. The clubmoss *L. annotinum* and the aquatic fern *S. natans* showed similar amounts of proteins compared with maize. All five of the terrestrial ferns (*D. filix‐mas*, *A. trichomanes*, *P. vulgare*, *P. aculeatum*, and *A. venustum*) contained from three‐ to 10‐fold higher amounts of proteins than maize plants (Figure 3).

#### 3.2.2 Total amino acids

**FIGURE 4:**
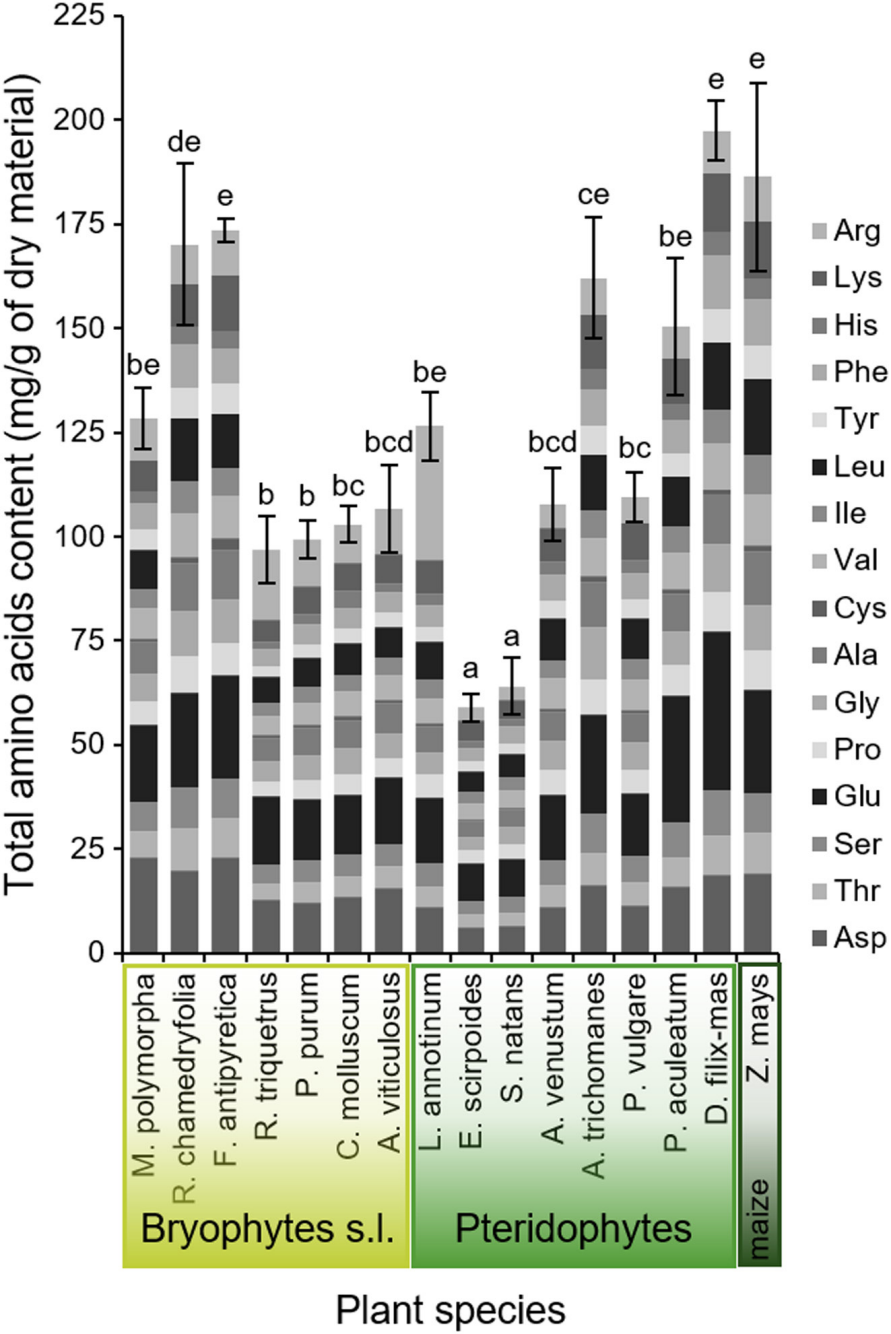
Total amino acid contents (total mean ± SE, *n* = 4 for each plant) quantified in primitive plants and maize. Tissue of one of the 7 bryophytic (light green), 8 pteridophytic (middle green), and maize (*Zea mays*, dark green) plants were analyzed. The statistics are based on the sum of every single amino acid content measured. Letters above the bars indicate statistical differences according to post‐ANOVA Tukey test results performed on log‐transformed data (ANOVA results: *F*: *F*_(15;48)_ = 17.22, *p*‐value < .001). For each species, the respective amounts of 16 amino acids are shown (see labels on the right side, as follows: Asp, Thr, Ser, Glu, Pro, Gly, Ala, Cys, Val, Ile, Leu, Tyr, Phe, His, Lys, and Arg).

In bryophytes, the liverworts *M. polymorpha* and *R. chamedryfolia*, as well as the aquatic moss *F. antipyretica*, had statistically similar total amino acid amounts as maize. In terrestrial mosses *R. triquetrus*, *P. purum*, *C. molluscum*, and *A. viticulosus*, significant lower amounts were measured (Figure 4). Total amino acid contents in mosses range typically from 100 to 170 mg/g of dry material (Figure 4), whereas protein amounts represent 5 to 10 mg/g of the dry material in this group (Figure 3). Three pteridophytic species offered a similar total amount with maize (around 160 m/g of dry material; *A. trichomanes*, *P. aculeatum*, and *D. filix‐mas*), two significantly less (around 110 mg/g of dry material, *A. venustum* and *P. vulgare*), and 2 offered the fewest quantities among all the plants tested (around 60 mg/g dry material; *E. scirpoides* and *S. natans*). Ferns, in contrast to their similar or higher amount in protein than the maize plants, showed total amino acid contents that were lower or similar to those of this seed plant (Figures 3 and 4). Based on the total amounts of amino acids only, ferns showed more diverse profiles pattern than mosses (Figure 4). When relative abundances of various amino acids were considered, profiles differ slightly between species, or significantly as for the clubmoss *L. annotinum* showing the largest proportion of arginine (Arg; Figure 4).

In a principal component analysis (PCA), including all the 16 different amino acids, species tended to spread out in a way that reflects the total amino acid contents. PCA was driven by species with high levels of amino acids on one side, whereas low amino acid species clustered together in the opposite direction (cf. Figure S1). High amounts of arginine (Arg) were detected in the clubmoss species *L. annotinum* explaining the intermediate position of this species within the pool (cf. Figure S2).

As well, relative amounts of amino acids were compared in both a perMANOVA and pairwise comparisons (cf. Table S1). This statistical approach improved the comparisons between amino acid profiles. PCA carried out on these relative amounts enabled the discrimination between most of the species, even though maize and five pteridophyte species (*E. scirpoides, S. natans, A. venustum, A. trichomanes*, and *P. vulgare*) showed little differences in amino acid proportions (cf. Figure S2).

**FIGURE 5:**
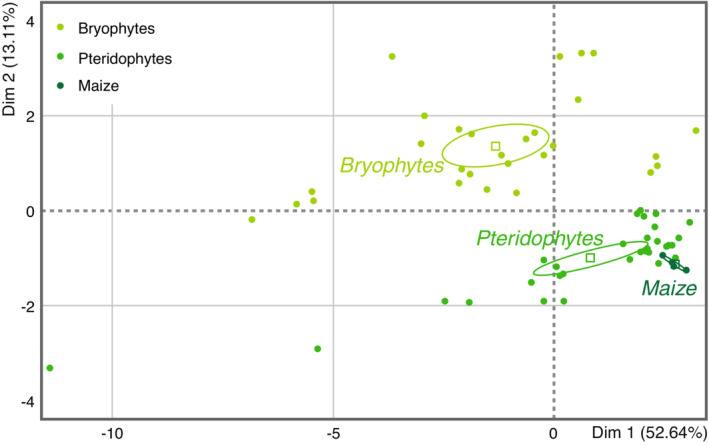
Principal component analysis clustering of the three upper plant groups studied, based on the relative amounts of 16 amino acids. The three taxa (with 95% confidence ellipses) include 16 species: 7 bryophytes (light green), 8 pteridophytes (middle green), and the additional maize plant (*Zea mays* in dark green).

The relative amounts of amino acids were also used in an overall PCA comparison between the main taxonomic groups (Figure 5). The nonvascular liverworts and mosses can be distinguished from the vascular pteridophytes (i.e., clubmoss, horsetail, and ferns) and maize plant, confirming differences in amino acid patterns.

#### 3.2.3 Free and storage sugars

**FIGURE 6:**
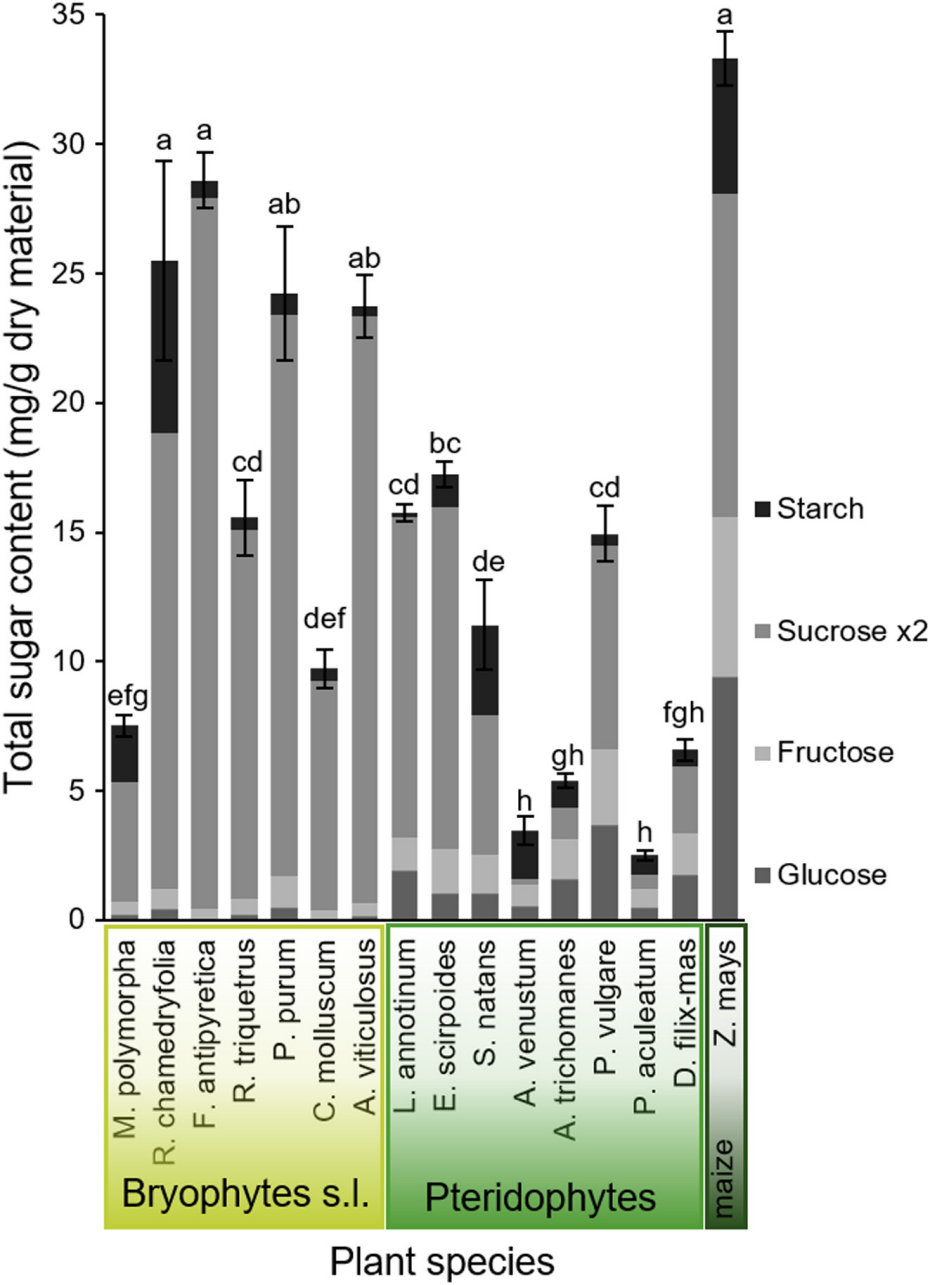
Total sugar contents (total mean ± SE, *n* = 4 for each plant) quantified in primitive plants and maize. Tissue of one of the 7 bryophytic (light green), 8 pteridophytic (middle green), and maize (*Zea mays*, dark green) plants were analyzed. The statistics are based on the sum of every single amino acid content measured. Letters above the bars correspond to post‐Kruskal–Wallis pair‐comparison tests (Bonferroni correction, *χ* ^2^ = 60.0, *p*‐value < .001).

**FIGURE 7:**
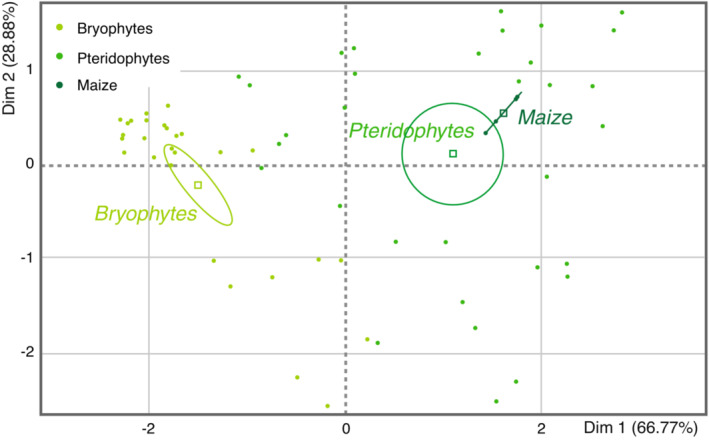
Principal component analysis based on relative amounts of sugars detected in mosses, ferns, and maize. The three upper groups (with 95% confidence ellipses) include 16 species: 7 bryophytes (light green), 8 pteridophytes (middle green), and the additional maize plant (*Zea mays* in dark green).

Sugars quantified in 16 species of bryophytes, pteridophytes, and maize varied strongly within a taxon (i.e., Hepaticophyta, Bryophyta, Lycophyta, Monilophyta). Four of the moss species had comparable levels of total sugars, whereas all pteridophytic species showed lower levels, as compared with maize (Figure 6). In order to assess properly the different quantities among the various sugar types (monosaccharides, disaccharides, polysaccharides), all quantities measured were transformed to fit with a single sugar unit taking the sugar monomers into account. The principal component analysis (PCA) carried out with these data reveals that fern profiles fall in between those measured for bryophytes and maize (Figure 7).

#### 3.2.4 Sugars amino acid ratios

**FIGURE 8:**
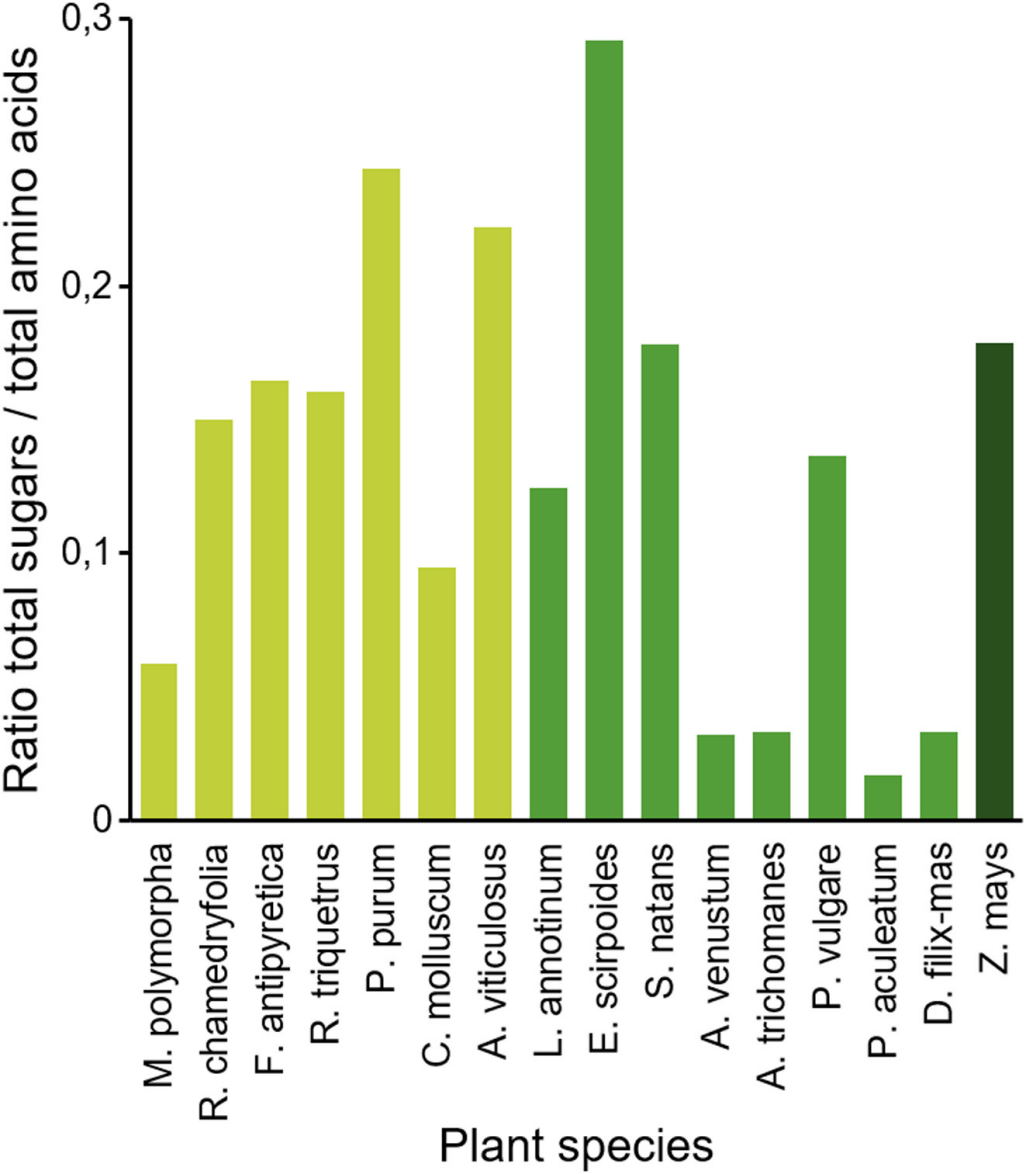
Sugar/amino acid ratios measured in primitive plants and maize. Tissue of one of the 7 bryophytic (light green), 8 pteridophytic (middle green), and maize (*Zea mays*, dark green) plants were analyzed.

In addition, we used the ratio between sugars and amino acid contents to identify whether some plant species provide unbalanced or unexpected profiles between these nutrients (Figure 8). Although sugar/amino acid ratios varied within both species and taxonomic groups, none of these profiles exhibited unusual patterns (Figure 8).

#### 3.2.5 Correlation between nutrient contents and developmental performances

**FIGURE 9:**
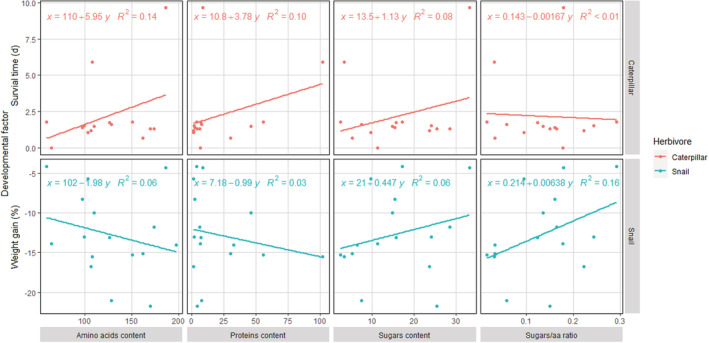
Linear regressions between nutrient parameters (amino acids, proteins, and sugar, all expressed in mg/g of dry tissue, and the sugar/amino acid ratio) measured in primitive plants, and the developmental performances of two generalist herbivores. The *R* ^2^ coefficients were calculated with the least square method.

Linear regressions of amino acids, proteins, sugar levels, and sugar/amino acid ratios in mosses and ferns against developmental performances of caterpillars and snails (i.e., survival time and weight gain, respectively) showed poor relationships (*R* ^2^ coefficient < 0.17, in all cases; Figure 9).

## 4 DISCUSSION

Overall, we found that the developmental performance and survival of the caterpillars and snails fed on early land plants were low. Both herbivores suffered negative consequences (i.e., mass loss, extended development time, and lack of development completion) when eating proposed bryophytes and pteridophytes. Under exact same experimental conditions, they performed, however, far better on the artificial diet, and to some extent on maize leaves, that we offered as control and alternative treatments, proving that diets alone can be responsible for the poor performances observed.

Usually able to complete all larval stages and metamorphosis in 18 days under favorable conditions, beet armyworm caterpillars (*S. exigua*) were critically disadvantaged in terms of development when fed on moss and fern leaves. Beyond the third week of the experiment, only one caterpillar fed on an early land plant was still alive (i.e., on the fern *A. venustum*), whereas 80% successfully finalized their development on artificial diet and 25% on maize. Maize plants appeared to not be an advantageous host for the polyphagous *S. exigua* caterpillars. Maize is a model organism showing well‐studied defensive mechanisms against herbivores (Qi et al., 2018). Here, maize was considered as an intermediate resource that herbivores may use in the field, defended but still suitable as a food supply for both caterpillars (Blanco et al., 2014) and snails (Barker, 2002). In this study, this plant cannot be seen as the inclusive spermatophyta representative in terms of nutrient contents and host suitability. As well, it is relevant to point out that before the feeding assays, all the caterpillars were fed with the nutritive artificial diet, probably contributing a part to the successful developmental rate gap observed between maize (25%) and this artificial diet (80%). Our findings confirm that moss and fern tissues are not an appropriate resource for a generalist insect, and why so few insects may exploit such plants in natural habitats. In 1980, Hendrix stated that the main fern‐feeding insects are specialized coleopteran, hemipteran, and lepidopteran species. In natural ecosystems, he observed that the number of insect feeders on ferns was far less than theoretically expected (Hendrix, 1980). We initially hypothesized that, in a no‐choice situation, the extremely polyphagous beet armyworm caterpillars with their powerful mouthparts should be readily able to feed on mosses or ferns (Azidah & Sofian‐Azirun, 2006; Saeed et al., 2019). Our observations support this hypothesis and caterpillars chew and ingest plant tissues, which was visually recorded but not quantified. These results predict that beyond likely preingestion deterrence, other mechanisms impairing digestion or assimilation may occur (Haines & Renwick, 2009).

For *C. barbara* snails, the mortality rate was low when fed on early land plants, but they only gained body mass when feeding on artificial diet (9% on average for 33 days). Maize is considered acceptable as food for snails and other terrestrial mollusk species (Barker, 2002), but the gastropod tested here lost on average 4% in weight during the feeding period on this plant. Although some of the plants had stronger negative effects than maize, most of the snails were not majorly disadvantaged when feeding on the leaves of bryophytes and pteridophytes. Above all, our results express the ability of snails to starve for many consecutive days, which allows them to avoid undesirable food. Under natural conditions, such behaviors have been observed during cold, hot, and dry periods (Boycott, 1934; Cáceres, 1997; Rees, 1964). Waiting for better conditions, snails are known to go into dormancy enclosed in their shell (Pomeroy, 1968). The snail avoidance towards these diets reinforces the notion that early land plants might not be suitable host plants. Previous long‐term experiments carried out on epiphytic cryptogam communities (lichens, algae, fungi, mosses) showed that gastropod grazing does not affect bryophytic diversity (Boch et al., 2016). In fact, spore dispersal by slugs may even promote bryophyte species diversity, suggesting positive outcomes of interactions between these plants and gastropods (Boch et al., 2015).

The qualitative and quantitative measurements of the nutrients provided by plant leaves failed to explain the poor performances by the herbivores. Compared with maize, concentrations of proteins in bryophytic species were either equivalent (2 species), or significantly lower (5 species). For pteridophytic leaves, they showed similar (2 species) or significantly higher concentrations (5 species), and only one of the species has a significantly lower amount. When correlating protein profiles with the developmental performance of *S. exigua* caterpillar or *C. barbara* snail, no causal connection emerged (Figure 9).

Amino acid contents showed variation between the studied plants, both in quantity and quality. Between the plant species, the amino acid patterns appeared to be mainly comparable, with few exceptions as for the clubmoss *L. annotinum* containing a higher amount of arginine. The importance of arginine in this species has not yet been studied and may warrant further investigations.

Paradoxically, if protein contents in mosses seem to be inferior to those found in pteridophytes and maize, their total amino acid contents showed to be equivalent or higher than those of many ferns. This could be explained by a high amount of free amino acids in mosses whereas pteridophytes and maize plants store these ones mainly inside their proteins. Free or fixed in proteins, none of our results suggests that amino acids may be responsible for the hypothetical poor nutritional quality of early land plants. Once again, when correlated with herbivore performance, no consistent patterns were found (Figure 9). The plant amino acid profiles, or ratios, can be of importance for insect growth (Bernays & Chapman, 1994, 2000) and interactions with plants, as shown for insect pollinators: butterflies favor nectar with high amino acid content, which drives the selection for nectar composition (Mevi‐Schütz & Erhardt, 2005). Similarly, amino acids in pollen have been shown to affect flower selection by solitary bees (Weiner et al., 2010). Based on our plants' nutritional values, generalist herbivores should cope with the varying amino acid and protein levels offered by ferns and mosses included in this study. Some specialized sap‐sucking aphids can develop on ferns, even though the phloem normally does not offer all of the required amino acids (Baumann, 2005). The natural fluctuations in amino acid availability and ratios can complicate feeding on mosses and ferns, but this is also the case with all other wild plants exploited by herbivores (Özcan, 2016; Watson & Creaser, 1975). To minimize possible constraints of varying nutrient contents, our plants were kept for two weeks in standard soil and under controlled conditions before feeding assays and nutrient quantifications. Interestingly, the principal component analyses based on amino acid profiles readily discriminated between the main plant taxa that were studied. Profiles for bryophytic species were distinct from those analyzed for both pteridophytes species and maize (Figure 5).

Both proteins and amino acids are of major importance for embryogenesis and the development of insect herbivores (Chen, 1966), then a lack of these primary metabolites in host plants could explain why herbivores are unable to successfully develop. As some proteins are involved in defense mechanisms, Markham et al. (2006) have suggested that their overall amounts may hardly be considered as a proper proxy for the effective nutritive quality of the plants. Nevertheless, as defensive proteins and many secondary metabolites in plants can be potent at very low levels (Felton, 1996; Machado et al., 2015), an overall protein content should still be a relevant estimation for dietary quality. When focusing on soluble proteins and digestible carbohydrates in seven Solanaceae and Martyniaceae species eaten by *Manduca sexta* moths (Sphingidae), Wilson et al. (2019) measured levels from 6.9 ± 5 to 15.6 ± 9.7% for soluble proteins. Despite different analytical procedures, the protein contents in our study, ranging from 1% to 12%, seem to show acceptable contents, and predicting that a major part of these ones can undoubtedly be considered as nutritive, including some initially involved in plant defenses but which could be overcome by herbivores (Bernays & Chapman, 1994, 2000). Based on a cautious interpretation of our results, this study confirms that early land plants offer appropriate global protein amounts, which contribute to dismiss the “lack of nutrients” hypothesis tested. As for most of the plants eaten by herbivores, the different functions of proteins in early land plants remain to be disentangled in order to know exactly which are nutritious.

The levels of free or stored sugars measured in the plants cannot explain the poor performance of polyphagous herbivores either. Half of the moss leaves contained sugar levels that were similar to those found in maize. By contrast, all the pteridophytic plants had significantly lower sugar quantities than maize (Figure 6). Just as for amino acids, overall sugar profiles were found to be taxon‐dependent, with bryophytic patterns distinctly separated from both ferns and maize (Figure 7). We found no correlation between plant sugar levels and herbivore growth or survival (Figure 9).

In addition to their energetic roles in plant metabolism, sugars are strongly involved in osmoregulation, especially in mosses, which show exceptional capacities to overcome drought (Bewley, 1979). Sucrose, which occurs in higher levels in mosses than in vascular plants, is particularly important as an osmotic regulator (Proctor, 2000; Smirnoff, 1992), and then as a protective agent against abiotic stress. Frequent desiccation of mosses might be a natural way to consistently challenge the herbivores, as it is difficult to specialize in a continuously changing resource. If so, sugars can be considered as both a nutritive resource and a protective agent in mosses. In the current study, however, this duality was not measurable, as only fresh and well‐hydrated plant material was used either for feeding or for chemical analyses.

By themselves, the amounts of amino acids or sugars in plants might not be entirely informative when determining their suitability for herbivores. We therefore also calculated sugar/amino acid ratios for each plant species (Figure 8). Regarding all the plants tested, these ratios cannot be linked as a factor explaining the reduced survival and mass lost in herbivores (Figure 9).

In this study, the notion that mosses and ferns offer reduced nutritive contents for herbivores is strongly refuted. Nevertheless, and paradoxically, our observations reinforce the view that generalist herbivores perform poorly when feeding on early land plants. Our results, as those of Haines and Renwick (2009), may potentially depend on the species of plants and herbivores chosen. Aiming to test the early plants’ low nutrient hypothesis, a clear focus on bryophytes and pteridophytes was dictated in this study, without ignoring that the addition of more angio‐ and gymnosperm species, as well as other herbivore types, would certainly contribute to have broader conclusions.

An alternative explanation for why current herbivores struggle to survive on these plants could be due to specific defense traits. Physical and chemical defenses must have evolved in these plants during their interactions with early herbivores. Physical defenses in primitive plants are considered weak, especially in mosses, which lack hard sclerenchyma tissues (Matsunaga et al., 2004). Current literature on mosses and ferns provides little information on physical traits that serve in a defensive context. Only trichomes of the aquatic fern *S. natans* have been well‐studied (Barthlott et al., 2009). However, these trichomes seem to be used more as flotation buoys than protective structures, as they appear not to deter aquatic herbivores that occasionally feed on these plants and others of the same genus (Tewari & Johnson, 2011). Physical defenses in bryophytes and pteridophytes await further scientific description and functional studies.

On the chemical side, certain defenses must have been involved in the early interactions between first land plants and newcomer herbivores. For bryophytes, some chemical compounds have been studied, mainly in liverworts and mostly from a medical application perspective. Liverworts are known to contain secondary metabolites in organelles called oil bodies, which were first described by Hübener (1834) but have only recently been studied from a chemical perspective, revealing lipophilic sesqui‐ and diterpenoids, phenolic compounds, and polyketides (Asakawa & Ludwiczuk, 2017; He et al., 2013). Chemicals produced by other mosses still need to be properly investigated. Above all, further investigations in this field should focus on the role of secondary metabolites in the plants' interactions with herbivores (Markham et al., 2006). Chemical compounds produced and released by pteridophytes have been slightly more studied. For instance, clubmosses (*Lycopodium* sp.) exhibit complex alkaloids (Aver & Trifonov, 1994; Kitajima & Takayama, 2011), and many chemical compounds produced by bracken ferns (i.e., *Pteridium* spp.) have been described (Jones & Firn, 1978; Schreiner et al., 1984). Cooper‐Driver (1978) studied insect‐fern associations and the role of secondary metabolites in the defense of bracken ferns against herbivory. It appears that they indeed contain defense compounds that have an impact on pathogens and herbivores (Agarwal et al., 2018).

If less relevant for pteridophytes, mosses frequently show small vegetative structures limiting their overall biomass in terrestrial ecosystems. De facto, these features could exclude many herbivores to exploit these plants, including large insects unable to finalize their development on such limited resources, and contributing to limit herbivores’ diversity on bryophytes. However, smaller invertebrates (e.g., Tardigrada) have been shown to perfectly develop on mosses. Possible damages in bryophytes by tiny herbivores would definitely require more attention.

Overall, it appears from our results that the attested dislike of herbivores for bryophytes and pteridophytes is not due to a lack of nutritional value but rather to not yet understood physical and chemical defenses, supporting the same conclusions as Haines and Renwick (2009). Therefore, follow‐up investigations on possible defensive mechanisms are needed. They may finally provide an explanation for the paradox of early land plants as a plentiful but neglected resource.

## 5 CONCLUSION

In controlled feeding experiments, it is shown that two polyphagous herbivores cannot properly develop and survive on 15 representative bryophytic and pteridophytic species. These findings confirm the standing notion that nonspecialized herbivores perform poorly and avoid feeding on such plants. The analyses of nutrient contents, however, suggest that the normally assumed explanation of poor nutritional value of bryophytes and pteridophytes is not what is responsible for the failure of the herbivores to develop on these plants. In terms of total proteins, amino acids, and sugar contents, the plants offer suitable amounts and concentrations, comparable to maize plants. The levels of these primary metabolites found in fresh leaves should be sufficient for the proper development and survival of both caterpillars and snails. Past assumptions that the bryophytes and pteridophytes' tissues are of poor nutrient quality should therefore be considered incorrect. Yet, as many early land plants are indeed unsuitable resources for herbivores, other factors must be involved. We predict that defensive mechanisms in bryophytes and pteridophytes are likely responsible for the poor performance and avoidance by herbivores.

## AUTHOR CONTRIBUTIONS

**Audrey Duhin:** Conceptualization (supporting); data curation (equal); investigation (lead); writing – original draft (equal). **Ricardo A. R. Machado:** Formal analysis (equal); writing – review and editing (equal). **Ted C. J. Turlings:** Conceptualization (equal); supervision (equal); writing – review and editing (equal). **Gregory Röder:** Conceptualization (equal); data curation (equal); formal analysis (equal); funding acquisition (lead); investigation (supporting); methodology (supporting); project administration (lead); supervision (lead); writing – original draft (equal); writing – review and editing (equal).

## FUNDING INFORMATION

This work was fully supported by the University of Neuchâtel.

## CONFLICT OF INTEREST

The authors have no conflict of interest to declare.

## Supporting information


Appendix S1

## Data Availability

All data supporting the manuscript are publicly available from the Dryad Digital Repository (https://doi.org/10.5061/dryad.4xgxd25dq).
