# Supplementary material for: Early land plants: Plentiful but neglected nutritional resources for herbivores?
Source: Ecol Evol. 2022 Dec 12;12(12):e9617. doi: 10.1002/ece3.9617 (PMC9745390; doi:10.1002/ece3.9617)
Supplement: Supplementary file 1 — Appendix S1 [file ECE3-12-e9617-s001.docx]

**Supplementary material**

| 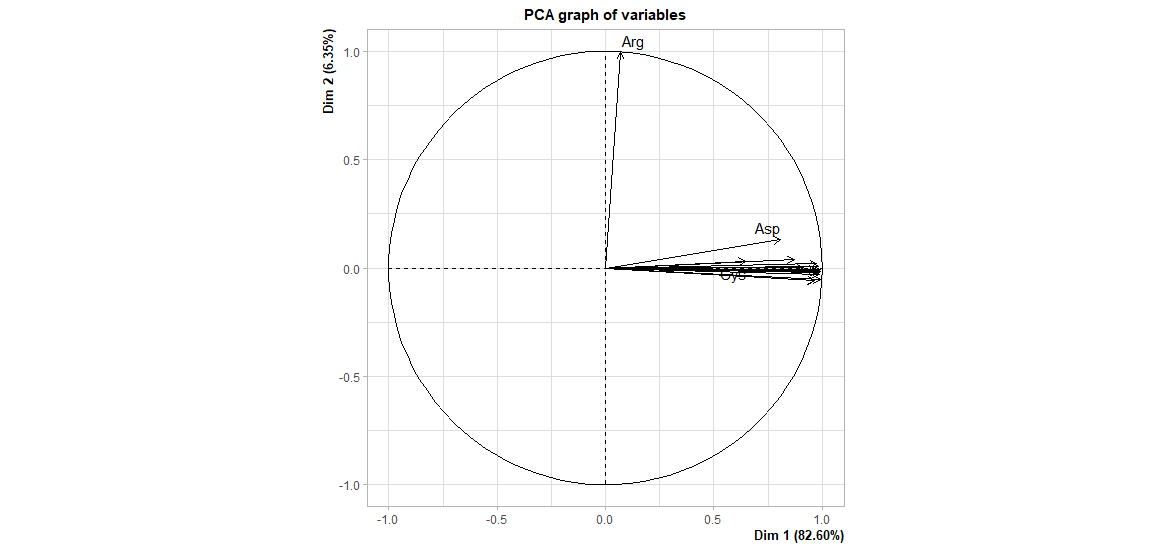  A  B  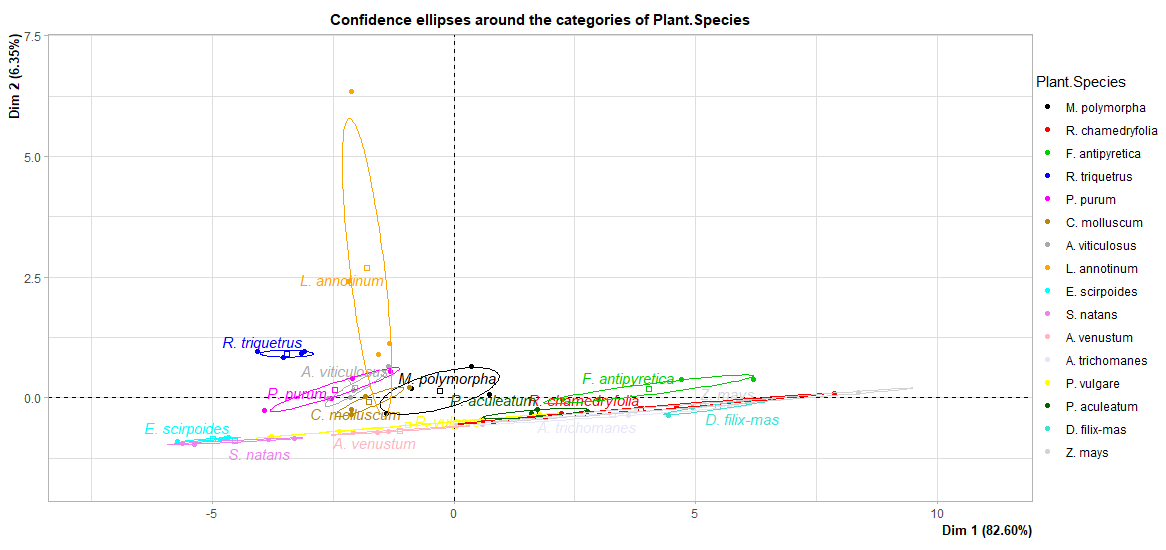 |
| --- |

Figure 1: Principal Component Analysis (PCA) based on the 16 amino acids quantified (n=4) in 15 early land plant (7 mosses and 8 ferns) and one angiosperm (maize) species, showing (A) the vectors formed by respective variables (amino acids) in the PCA projection and (B) the confident ellipses (95%) formed around the plant species.

| 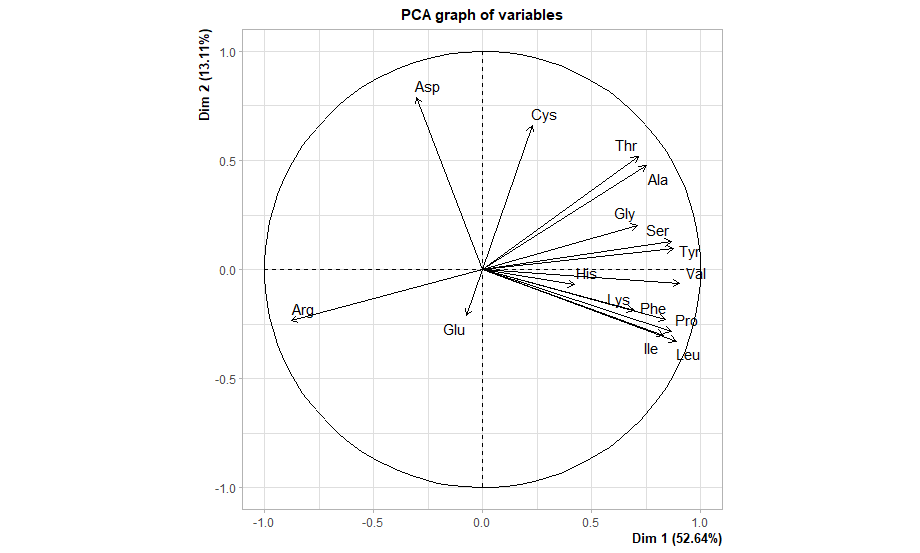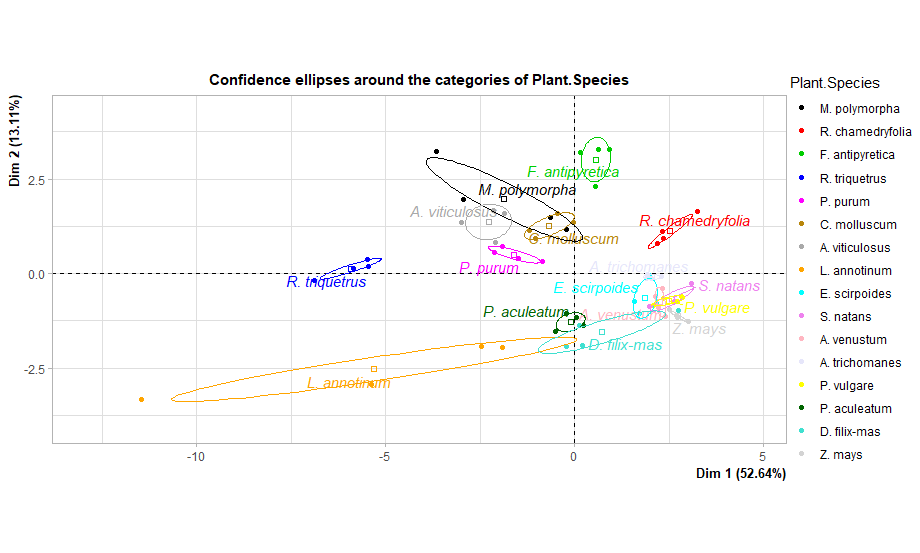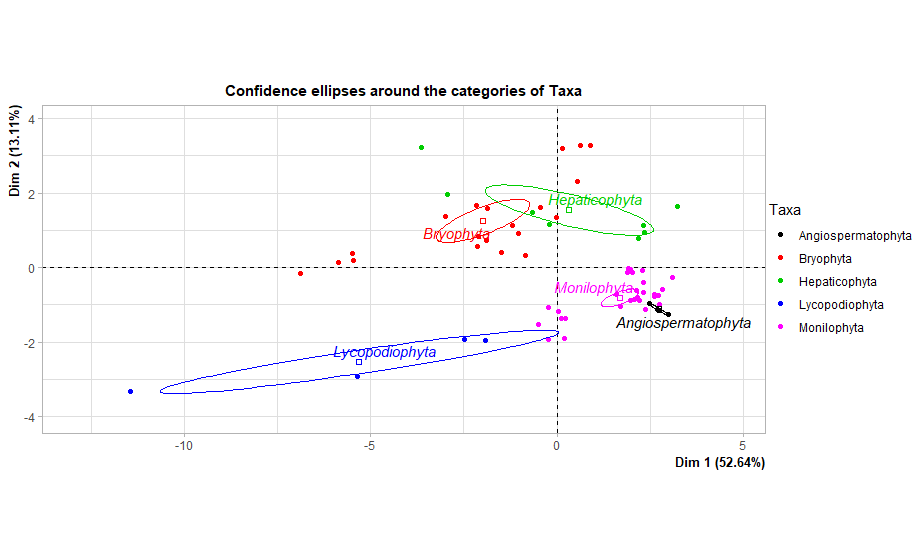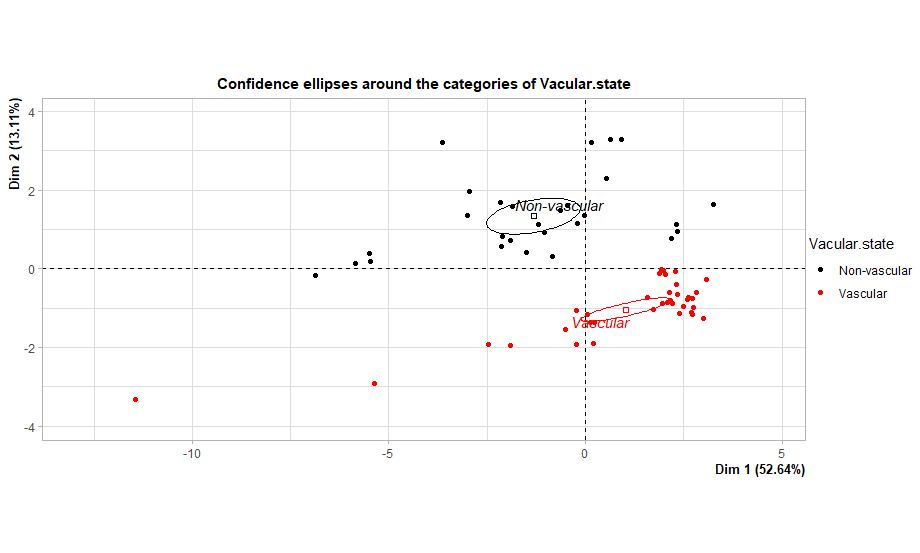  B  D  C  A |
| --- |

Figure 2: PCA based on the relative amounts of 16 amino acids quantified (n=4) in 15 primitive plants, including 7 Bryophytes, 8 Pteridophytes and one angiosperm (maize) showing (A) the vectors formed by the originate variables in the PCA projection; the confident ellipses (95%) formed around (B) the plant species, (C) plant taxa and (D) plant vascular-state group.

Table 1: PerMANOVA and pairwise comparisons on amino acids contents results. The post-perMANOVA performed (1) is reported in the bottom left triangle on rough data, and (2) in the upper right triangle on relative amount. The perMANOVA was performed with adonis function (999 permutations, Bray-Curtis distance method). Except in gray cells, all pairs significantly differ (p < 0.05).

| Species | *M. polymorpha* | *R. chamedryfolia* | *F. antipyretica* | *R. triquetrus* | *P. purum* | *C. molluscum* | *A. viticulosus* | *L. annotinum* | *E. scirpoides* | *S. natans* | *A. venustum* | *A. trichomanes* | *P. vulgare* | *P. aculeatum* | *D.  filix-mas* | *Z. mays* |
| --- | --- | --- | --- | --- | --- | --- | --- | --- | --- | --- | --- | --- | --- | --- | --- | --- |
| *M. polymorpha* |  | 0.018 | 0.025 | 0.012 | 0.006 | 0.018 | 0.028 | 0.001 | 0.026 | 0.026 | 0.019 | 0.015 | 0.025 | 0.017 | 0.011 | 0.024 |
| *R. chamedryfolia* | 0.034 |  | 0.031 | 0.024 | 0.017 | 0.031 | 0.022 | 0.005 | 0.032 | 0.032 | 0.03 | 0.026 | 0.022 | 0.02 | 0.015 | 0.031 |
| *F. antipyretica* | 0.001 | 0.659 |  | 0.006 | 0.001 | 0.017 | 0.005 | 0.003 | 0.009 | 0.004 | 0.008 | 0.016 | 0.02 | 0.001 | 0.001 | 0.003 |
| *R. triquetrus* | 0.015 | 0.001 | 0.008 |  | 0.001 | 0.001 | 0.008 | 0.003 | 0.022 | 0.006 | 0.001 | 0.001 | 0.007 | 0.004 | 0.001 | 0.001 |
| *P. purum* | 0.014 | 0.001 | 0.028 | 0.095 |  | 0.023 | 0.009 | 0.001 | 0.032 | 0.017 | 0.022 | 0.018 | 0.015 | 0.018 | 0.023 | 0.022 |
| *C. molluscum* | 0.019 | 0.001 | 0.001 | 0.001 | 0.451 |  | 0.016 | 0.01 | 0.009 | 0.007 | 0.003 | 0.001 | 0.009 | 0.001 | 0.004 | 0.001 |
| *A. viticulosus* | 0.055 | 0.001 | 0.023 | 0.001 | 0.314 | 0.118 |  | 0.001 | 0.024 | 0.027 | 0.018 | 0.019 | 0.036 | 0.029 | 0.019 | 0.03 |
| *L. annotinum* | 0.001 | 0.001 | 0.001 | 0.001 | 0.001 | 0.014 | 0.001 |  | 0.017 | 0.02 | 0.024 | 0.028 | 0.02 | 0.022 | 0.025 | 0.02 |
| *E. scirpoides* | 0.018 | 0.001 | 0.006 | 0.02 | 0.028 | 0.001 | 0.014 | 0.017 |  | 0.006 | 0.012 | 0.001 | 0.005 | 0.012 | 0.001 | 0.012 |
| *S. natans* | 0.016 | 0.001 | 0.006 | 0.007 | 0.048 | 0.003 | 0.011 | 0.014 | 0.519 |  | 0.013 | 0.014 | 0.001 | 0.001 | 0.085 | 0.016 |
| *A. venustum* | 0.039 | 0.001 | 0.006 | 0.001 | 0.037 | 0.029 | 0.001 | 0.034 | 0.035 | 0.022 |  | 0.032 | 0.806 | 0.005 | 0.006 | 0.024 |
| *A. trichomanes* | 0.037 | 0.607 | 0.303 | 0.005 | 0.001 | 0.012 | 0.008 | 0.021 | 0.02 | 0.014 | 0.01 |  | 0.013 | 0.027 | 0.026 | 0.017 |
| *P. vulgare* | 0.232 | 0.027 | 0.001 | 0.001 | 0.4 | 0.478 | 0.385 | 0.024 | 0.023 | 0.037 | 0.004 | 0.066 |  | 0.036 | 0.003 | 0.001 |
| *P. aculeatum* | 0.001 | 0.167 | 0.001 | 0.001 | 0.001 | 0.017 | 0.001 | 0.017 | 0.023 | 0.019 | 0.001 | 0.272 | 0.009 |  | 0.006 | 0.001 |
| *D. filix-mas* | 0.011 | 0.19 | 0.05 | 0.006 | 0.001 | 0.01 | 0.002 | 0.033 | 0.012 | 0.02 | 0.099 | 0.014 | 0.016 | 0.003 |  | 0.009 |
| *Z. mays* | 0.062 | 0.631 | 0.459 | 0.007 | 0.004 | 0.009 | 0.004 | 0.015 | 0.021 | 0.009 | 0.027 | 0.362 | 0.07 | 0.127 | 0.429 |  |
